# Functional signaling and gene regulatory networks between the oocyte and the surrounding cumulus cells

**DOI:** 10.1186/s12864-018-4738-2

**Published:** 2018-05-10

**Authors:** Fernando H. Biase, Katelyn M. Kimble

**Affiliations:** Department of Animal Sciences, Auburn University, 559 Devall Dr, Auburn, AL 36849 USA

**Keywords:** Inter-cellular communication, Oocyte-cumulus signaling, Gametogenesis, Gene regulatory networks

## Abstract

**Background:**

The maturation and successful acquisition of developmental competence by an oocyte, the female gamete, during folliculogenesis is highly dependent on molecular interactions with somatic cells. Most of the cellular interactions identified, thus far, are modulated by growth factors, ions or metabolites. We hypothesized that this interaction is also modulated at the transcriptional level, which leads to the formation of gene regulatory networks between the oocyte and cumulus cells. We tested this hypothesis by analyzing transcriptome data from single oocytes and the surrounding cumulus cells collected from antral follicles employing an analytical framework to determine interdependencies at the transcript level.

**Results:**

We overlapped our transcriptome data with putative protein-protein interactions and identified hundreds of ligand-receptor pairs that can transduce paracrine signaling between an oocyte and cumulus cells. We determined that 499 ligand-encoding genes expressed in oocytes and cumulus cells are functionally associated with transcription regulation (FDR < 0.05). Ligand-encoding genes with specific expression in oocytes or cumulus cells were enriched for biological functions that are likely associated with the coordinated formation of transzonal projections from cumulus cells that reach the oocyte’s membrane. Thousands of gene pairs exhibit significant linear co-expression (absolute correlation > 0.85, FDR < 1.8 × 10^− 5^) patterns between oocytes and cumulus cells. Hundreds of co-expressing genes showed clustering patterns associated with biological functions (FDR < 0.5) necessary for a coordinated function between the oocyte and cumulus cells during folliculogenesis (i.e. regulation of transcription, translation, apoptosis, cell differentiation and transport).

**Conclusion:**

Our analyses revealed a complex and functional gene regulatory circuit between the oocyte and surrounding cumulus cells. The regulatory profile of each cumulus-oocyte complex is likely associated with the oocytes’ developmental potential to derive an embryo.

**Electronic supplementary material:**

The online version of this article (10.1186/s12864-018-4738-2) contains supplementary material, which is available to authorized users.

## Background

During folliculogenesis, the interaction between an oocyte and the surrounding somatic cells evolves with the release of the oocyte from quiescence, through ovulation and fertilization, to zygote formation. As folliculogenesis progresses and a cavity is formed in the follicle, the somatic cells surrounding the oocytes, namely, granulosa cells, differentiate into cumulus and mural granulosa cells [1], and the cumulus-oocyte complex (COC) is formed. The proximity between the cumulus cells and the oocyte favors bidirectional communication, which is paramount for the acquisition of developmental competence by the oocyte.

In the microenvironment of an antral follicle, the cellular communication between oocytes and cumulus cells is complex, and both sides have active regulatory roles. The cumulus cells support meiotic arrest and cytoplasmic maturation of the oocyte, for example, by exporting cyclic AMP [2], calcium [3], other metabolites [4, 5] and unknown signals that control transcription in the enclosed oocyte [6]. The oocyte secretes growth factors that promote cumulus cell differentiation and proliferation [4] and maintain their differentiated state, preventing their transition to mural granulosa cells [7]. Most of these compounds are exchanged through gap junctions that connect their membranes [8]. Macromolecules, such as RNAs [9, 10], can also be transported from cumulus cells to oocytes; nonetheless, the specific functions and mechanism of transport are not yet understood.

The transcriptome profile of cumulus cells [11–19] and oocytes [20–24] reflects the developmental potential for successful fertilization and embryo formation. Nevertheless, little is known regarding the connection between the genes expressed in oocytes and cumulus cells. In this study, we aimed to determine the gene regulatory networks in COCs in two dimensions: (i) within each compartment and (ii) between oocytes and the surrounding cumulus cells. The results provide evidence that the communication between oocyte and cumulus cells in the follicle is complex, involving signaling through several ligand-receptor pairs and regulation of thousands of genes and is functionally relevant for the acquisition of oocyte developmental competence.

## Results

### Transcriptome profiling of single oocytes and corresponding cumulus cells

**Fig. 1:**
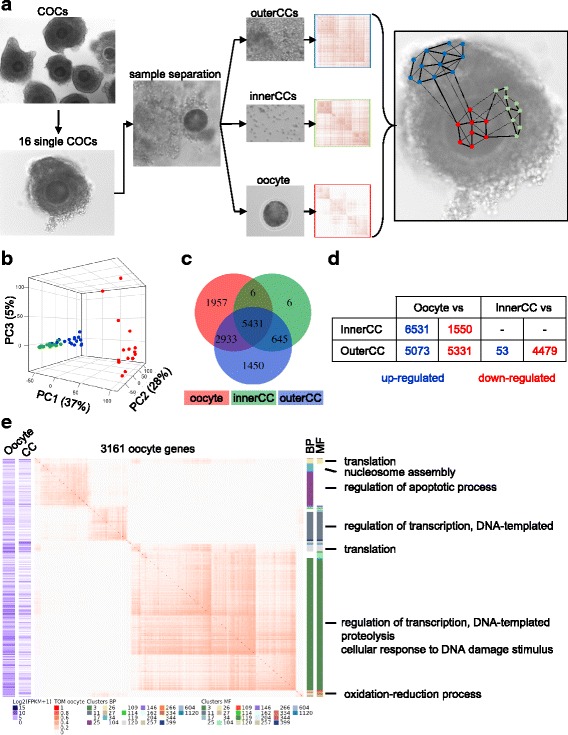
Transcriptome heterogeneity between oocytes and surrounding cumulus cells. (**a**) Depiction of the experimental design and the sequenced samples. (**b**) Principal component analysis of 12,482 genes quantified in oocytes (red), innerCCs (green) and outerCCs (blue). (**c**) Overlap of genes expressed in oocytes (red), innerCCs (green) or outerCCs (blue). (**d**) Differentially expressed genes determined from the pairwise comparisons of the three sample types (FDR < 0.01). (**e**) Co-expression analysis of genes expressed in oocytes showing differential expression compared those in cumulus cells (CC). The blue heatmaps depict the average expression in oocytes and CCs; the center heatmap depicts the topological overlap of correlated expression; and the vertical bars show the annotation of GO biological processes (BP) and molecular functions (MF) enriched in the corresponding gene cluster. The squares below the heatmap provide a link between cluster number, the corresponding biological process or molecular functions and genes presented in the supplementary (Additional file 3: Tables S8-S9)

We profiled the transcriptomes of 16 individual COCs collected *ex vivo* from bovine ovaries (*Bos taurus*). In each COC, we chemically dissected the outer layer of cumulus cells (outerCCs), the layers of cumulus cells closest to the zona pelucida (innerCCs), and the oocyte (Fig. 1a). Using the SMART-Seq2 approach, we generated over 390.4 million paired-end reads, with an average of 10.6, 6.2 and 7.4 million fragments obtained for single oocytes, innerCCs and outerCCs, respectively. Overall, we quantified the transcript levels of 19,847 genes, and we carried out analytical procedures for the genes presenting fragments per kilobase per million (FPKM) > 0.5 in more than eight samples (Additional file 1: Figure S1). Overall, we detected the transcripts of 10,327, 6088 and 10,459 genes in oocytes, innerCCs and outerCC cells, and a total of 12,482 genes were robustly quantified in COCs.

### Distinct functional transcriptome profiles of single oocytes and corresponding cumulus cells

Principal component analysis of the transcriptome data confirmed the expected distinction between the transcript profiles of oocytes and cumulus cells (*P* < 2.81 × 10^− 28^, Fig. 1b, 1000 randomizations, Additional file 1: Figure S2). Surprisingly, however, the second major source of variability (~ 28%) derived from the cumulus samples separated most of the innerCCs and outerCCs into two distinct groups (*P* = 2.81 × 10^− 28^, Fig. 1b, Additional file 1: Figure S2). Examination of the genes expressed in each of the three sample types collected from COCs showed that 5431 genes were expressed in oocytes and cumulus cells, while 1957, 1450 and 6 genes were exclusively expressed in oocytes (i.e. bone morphogenetic protein 4, bone morphogenetic protein 6, bone morphogenetic protein 15, folliculogenesis specific bHLH transcription factor, growth differentiation factor 9, Y-box binding protein 2), outerCCs (i.e. androgen receptor, cell adhesion molecule 4, estrogen receptor, fibroblast growth factor 11, insulin receptor) and innerCCs (i.e: olfactory receptor 6C74, olfactory receptor 12D2, and the potential novel genes: ENSBTAG00000038961, ENSBTAG00000045654, ENSBTAG00000046868, ENSBTAG00000046958), respectively (Fig. 1c, see Additional file 2 for full gene list with average expression data). The clustering of the two distinct groups of cumulus cells (outerCCs and innerCCs) was a strong indication of distinct functions associated with their spatial proximity to the oocyte.

We then used a co-expression framework and performed Gene Ontology (GO) enrichment testing of gene clusters [25] to reveal the functional patterns of gene co-regulation in each of the three sample types collected. In oocytes, there were 2222 co-expressed genes forming 26 clusters with significant enrichment of several GO terms (FDR < 0.2, Additional file 1: Figure S3, Additional file 3: Tables S1-S2). The biological function with the greatest number of annotated genes was “regulation of transcription” (69 genes in clusters 12, 31, 111 and 331, i.e. *SALL4*, *SFPQ*, *SIX3*, *SMAD4*, *YBX1*, *ZNF34*) followed by “translation” (54 genes in clusters 5, 234, 405 and 416, i.e. *AIMP2*, *EIF1*, *EIF2B2*, *EIF3I*, *EIF3K*). In innerCCs, we identified 1222 expressed genes forming six clusters with significant enrichment of GO terms (FDR < 0.2, Additional file 1: Figure S4, Additional file 3: Tables S3-S4). Eighty-one genes in cluster four were annotated with the biological process “translation”. The second most-represented term was “oxidation-reduction process” in cluster 178, with ten annotated genes. In outerCCs, we identified 3990 expressed genes distributed in 34 clusters with significant enrichment of GO terms (FDR < 0.2, Additional file 1: Figure S5, Additional file 3: Tables S5-S6). There were 92 genes in clusters 1, 37, 53 and 5504 annotated with the term “oxidation-reduction process”. Sixty-five genes in clusters 22, 32, 94 and 160 were associated with “translation”. The next most-represented function was related to transport, with 31 genes distributed among the terms “transport,” in cluster 22, and “vesicle-mediated transport” and “ER to Golgi vesicle-mediated transport,” in cluster 6. The results indicated distinct regulatory gene wiring of cumulus cells that are closer to or farther from the oocyte, which likely reflects their functional relevance to oocyte maturation during folliculogenesis.

Quantitative comparison of gene levels between samples revealed hundreds of differentially expressed genes (FDR < 0.01, Fig. 1d, Additional file 1: Figure S6) and further supported the distinction of the transcriptome profiles between oocytes and cumulus cells and between cumulus cells at different positions relative to the oocyte. Compared with their expression in cumulus cells, approximately 92% of the genes expressed in oocytes presented significant differential expression (9489/10,327, FDR < 0.01, Fig. 1d). We selected five genes that were overexpressed in oocytes and have previously been associated with developmental competence in oocytes (*ENY2*, *FSD1*, *GHR*, *METTL18* and *MYF6*) to validate the RNAseq results. The qPCR results showed that all the genes presented higher expression levels in oocytes than in cumulus cells (*P* < 0.05, Additional file 3: Table S7).

We further performed co-expression analysis of the transcript levels of these 9489 genes in oocytes and identified 31161 genes forming 16 and 19 clusters of co-expressed genes enriched (FDR < 0.2) for GO terms. There were 165 genes across five co-expression clusters (3, 11, 114, 257, 399) associated with the regulation of transcription (FDR < 0.2, Fig. 1e, Additional file 3: Tables S8 and S9). The second most-represented term was “proteolysis,” with 45 annotated genes in cluster 3, followed by “cellular response to DNA damage stimulus,” with 32 and three genes in clusters 3 and 146, respectively. The fourth most-represented term was “negative regulation of apoptotic process,” with 30 genes annotated in cluster 3. The results showed that the functional differences between oocytes and cumulus cells are mostly related to the regulation of transcription, proteolysis and apoptosis.

### Road map of ligands and receptors between oocytes and surrounding cumulus cells

The signaling between the oocyte and cumulus cells is bidirectional and is mediated in part by ligands and receptors [26]. We examined the expression of genes encoding ligand-receptor pairs supported by experimental evidence compiled from four protein-protein interaction databases. Using Ensembl gene homology annotation for human, mouse, pig, rabbit and rat, we identified 247,064 unique protein-protein interactions (PPIs) with corresponding cow Ensembl genes.

After integration of the transcriptome data with the PPI network, we identified 5226 and 192 genes expressed in COCs corresponding to ligand and receptor proteins, respectively, with the potential to form 14,011 interactions (Table 1, see Additional file 4 for detailed annotation of this dataset). Among the interacting proteins previously validated experimentally we identified in our dataset GDF9 - BMPR2, INHA-ACVR1, INHA-ACVR2B, indicating the validity of our approach.

**Table 1:** Summary of potential ligand-receptor interactions in cumulus-oocyte complexes and corresponding genes expressed in oocytes and cumulus cells annotated

Type of signaling	N^a^ pairs (PPI^b^)	N genes expressed			
		Ligand		Receptor	
		Oocyte	CC	Oocyte	CC
Autocrine/Paracrine	964	–	–	131	134
Autocrine	52	–	–	24	–
Autocrine	40	–	–	–	21
Autocrine/Paracrine	5489	2463	2463	93	93
Autocrine/Paracrine	546	268	–	81	81
Autocrine/Paracrine	860	–	397	86	86
Autocrine/Paracrine	1050	778	778	51	–
Autocrine/Paracrine	3762	2790	2790	–	42
Autocrine	146	114	–	35	–
Autocrine	566	–	442	–	31
Paracrine	200	–	156	39	–
Paracrine	336	262	–	–	31

^a^*N* number, ^b^*PPI* protein-protein interaction

**Fig. 2:**
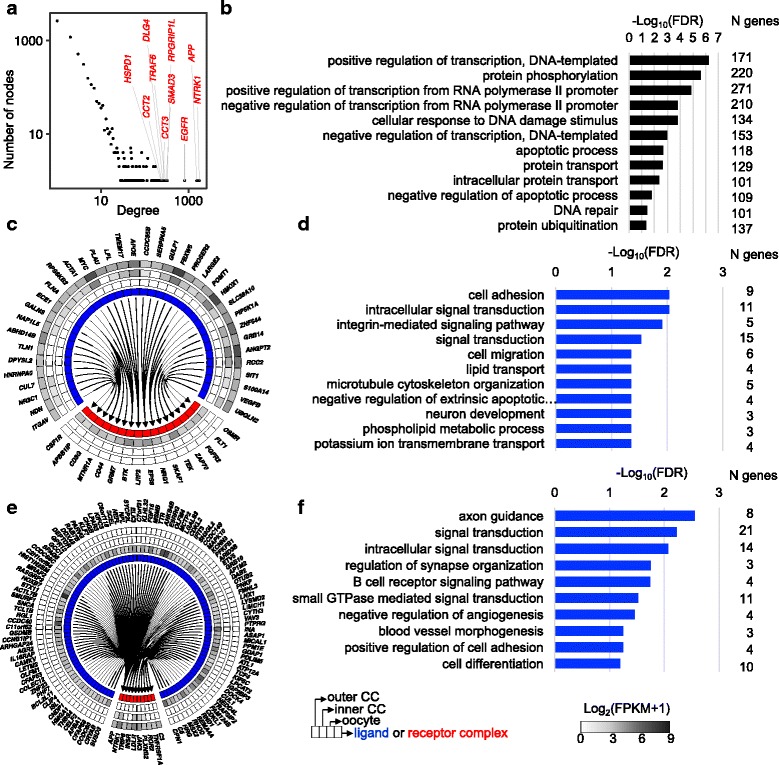
Ligand-receptor pairs in oocytes and surrounding cumulus cells. (**a**) Distribution of ligands or receptors among genes expressed in COCs according to the degree of connectivity. (**b**) Biological processes enriched (FDR < 0.05) in ligands or receptors in coding genes expressed in COCs (see Additional file 3: Table S13 for full list of terms). (**c**) Ligand-encoding genes expressed in CCs with receptor-encoding genes expressed in oocytes. (**d**) Biological processes enriched (FDR < 0.05) in ligand-encoding genes expressed in CCs with receptor-encoding genes expressed in oocytes. (**e**) Ligand-encoding genes expressed in oocytes with receptor-encoding genes expressed in CCs. (**f**) Biological processes enriched (FDR < 0.05) in ligand-encoding genes expressed in oocytes with receptor-encoding genes expressed in CCs. To improve readability, genes with average FPKM > 2 were plotted on panels **c** and **e**

Using the expression data for these genes encoding ligands and receptors, we distinguished the following categories of potential ligand-receptor interactions between oocytes and cumulus cells: 12,671 autocrine or paracrine, 804 autocrine and 536 paracrine (Table 1). The PPIs inferred for COCs formed a scale-free network (R^2^ = 0.65, *P* = 0.014, 1000 bootstrap simulations) with ten genes (*APP*, *CCT2*, *CCT3*, *DLG4*, *EGFR*, *HSPD1*, *NTRK1*, *RPGRIP1L* and *TRAF6*), accounting for ~ 18% of the ligand-receptor interactions (Fig. 2a).

Functional interrogation of the genes expressed in COCs encoding ligands and receptors with the potential to form PPIs revealed dozens of genes with significant enrichment in GO terms related to the “regulation of transcription” (*N* = 499), “protein phosphorylation,” “apoptotic process” and “protein transport” categories (FDR < 0.05, Fig. 2b, Additional file 3: Table S10). We then focused on the 156 ligand-encoding genes that were exclusively expressed in cumulus cells with corresponding receptor-encoding genes exclusively expressed in oocytes (Fig. 2c). These 156 ligand-encoding genes were enriched for “cell adhesion,” “signal transduction,” and “cell migration,” among other biological processes (FDR < 0.1, Fig. 2d, Additional file 3: Table S11). We also queried the 262 ligand-encoding genes that were exclusively expressed in oocytes with corresponding receptor-encoding genes exclusively expressed in cumulus cells (Fig. 2e). Among the significantly enriched processes, we noted “signal transduction,” “cell differentiation” and “axon guidance” as the terms with largest numbers of genes (FDR < 0.1, Fig. 2f, Additional file 3: Table S12). Taken together these results support the notion that ligand-receptor interactions are a major contributor to the signaling between oocytes and cumulus cells.

### Variation in gene expression associated with developmental competence

**Fig. 3:**
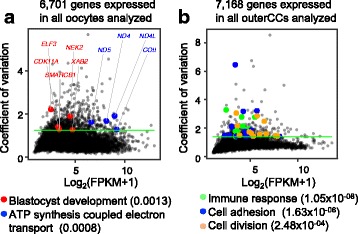
Variability in gene expression in COCs associated with biological functions. Coefficients of variation for genes expressed in all samples of oocytes (**a**) and outerCCs (**b**). Genes associated with specific categories are indicated with colored dots (*P*-value). The green line indicates the coefficient of variation threshold used to identify highly variable genes

The sampled COCs represented variable maturing stages of antral follicles, and we hypothesized that physiological changes in the growing antral follicles would result in variable expression of genes functionally related to developmental competence in oocytes and cumulus cells. Focusing on the 6701 and 7168 genes expressed in all 16 analyzed oocytes and outerCCs, we observed median coefficients of variance (CV) of 0.66 and 0.81, respectively. Functional investigation of the top variable genes (*N* = 670 genes, CV > 1.2, 90th percentile) revealed a tendency for enrichment (FDR = 0.3) of the GO terms “blastocyst development” (*CDK11A*, *ELF3*, *NEK2* and *SMARCB1*) and “ATP synthesis coupled electron transport” (Fig. 3a). Interrogation of the top variable genes in outerCCs (*N* = 359 genes, CV > 1.4, 95th percentile) highlighted several significantly enriched (FDR < 0.05) GO terms, with the top three terms being “immune response,” “cell adhesion,” and “cell division” (Fig. 3b, Additional file 3: Table S13).

### Co-regulated gene expression between the oocyte and corresponding cumulus cells

Analysis of three samples from the same COC allowed us to explore gene co-expression between oocytes and cumulus cells. We calculated the biweighted correlation (bicor) values for genes expressed in oocytes and innerCCs and for genes expressed in oocytes and outerCCs. Several pairs of genes showed significantly correlated expression between oocytes and cumulus cells (empirical (e) FDR < 0.001, 10,000 randomizations, Additional file 1: Figure S7a). The numbers of genes showing a correlation between oocytes and outerCCs or oocytes and innerCCs were not predictive of one another (*P* < 6 × 10^− 05^, Fisher’s exact test, Additional file 1: Figure S7b). Furthermore, there was little overlap between the genes when we examined a specific correlation threshold (|bicor| > 0.85, Additional file 1: Figure S7c), providing further support for the hypothesis that the regulatory interaction is influenced by the specific localization of the cumulus cells relative to the oocyte.

**Fig. 4:**
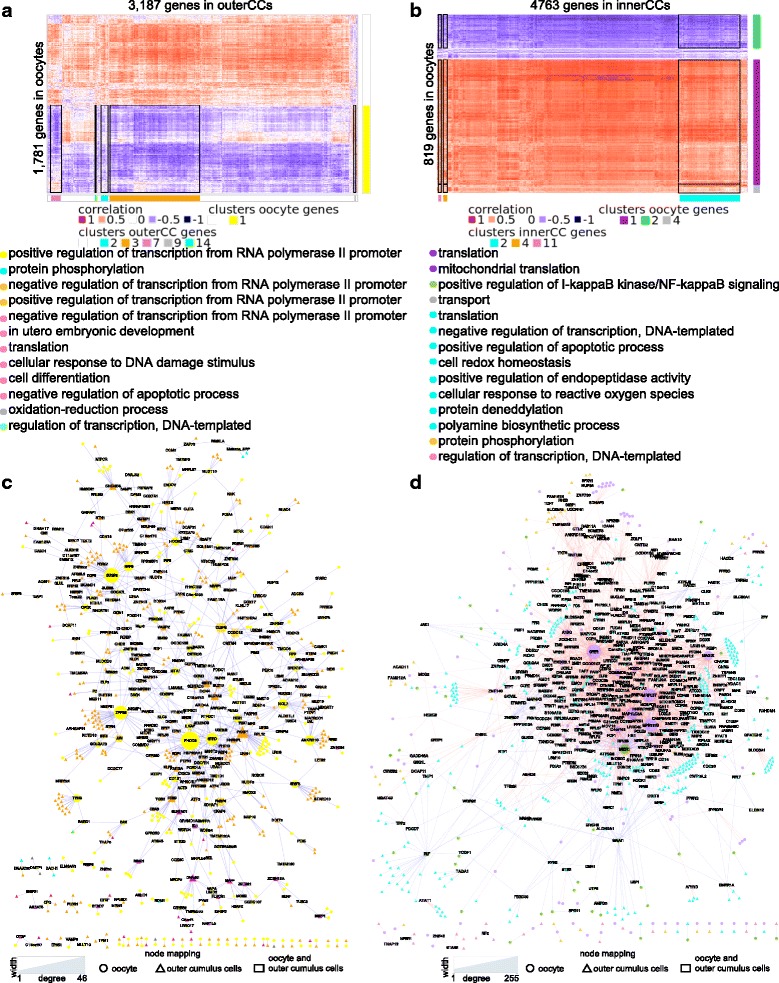
Functional co-expression between oocytes and surrounding cumulus cells. Heatmap of genes showing a high correlation (|bicor| > 0.85) of expression values between oocytes and outerCCs (**a**) and oocytes and innerCCs (**b**). Horizontal and vertical bars next to the heatmaps annotate gene clusters with enriched biological processes (FDR < 0.2). The black boxes indicate the co-expression blocks with intersection of clusters enriched for biological processes. Co-expression networks between oocytes and outerCCs (**c**) and oocytes and innerCCs (**d**). The color of symbols in panels c and d correspond to the colored circles and the GO biological processes on panels **a** and **b**, respectively

We then focused on the 7034 pairs of genes expressed in oocytes (*N* = 1781) and outerCCs (*N* = 3187) that presented a strong positive or negative correlation (|bicor| > 0.85, eFDR< 1.8 × 10^− 5^) and asked whether the genes could be clustered according to their biological function. We used the WGCNA approach to independently cluster the 1781 and 3187 genes expressed in oocytes and outerCCs, respectively. The 1781 genes expressed in oocytes formed one co-expression cluster (*N* = 57 annotated genes) enriched for “positive regulation of transcription from RNA polymerase II promoter”. The 3187 genes expressed in outerCCs formed five co-expression clusters enriched for biological processes (FDR < 0.05, Fig. 4a), including “protein phosphorylation” in cluster 2; “negative regulation of transcription from RNA polymerase II promoter” and “positive regulation of transcription from RNA polymerase II promoter” in cluster 3; “negative regulation of transcription from RNA polymerase II promoter,” “in utero embryonic development,” “translation,” “cellular response to DNA damage stimulus,” “cell differentiation” and “negative regulation of apoptotic process” in cluster 7; “oxidation-reduction process” in cluster 9; and “regulation of transcription, DNA dependent” in cluster 14.

There were 42,843 pairs of genes expressed in oocytes (*N* = 819) and innerCCs (*N* = 4569) that presented a strong positive or negative correlation (|bicor| > 0.85, eFDR < 1.8 × 10^− 5^). Co-expression analysis of the 819 genes expressed in the oocytes formed three clusters enriched for biological processes (FDR < 0.05, Fig. 4a): “translation” and “mitochondrial translation” in cluster 1; “positive regulation of I-kappaB kinase/NF-kappaB signaling” in cluster 2; and “transport” in cluster 4. The selected genes expressed in innerCCs formed three co-expression clusters enriched for biological processes (FDR < 0.05, Fig. 4b): “translation,” “negative regulation of transcription, DNA-templated,” and “positive regulation of apoptotic process,” among others in cluster 2; “protein phosphorylation” in cluster 4; and “regulation of transcription, DNA-templated” in cluster 11.

The clustering of the genes with correlated expression revealed patterns across genes with different functionalities (Fig. 4 a, b). For example, cluster 1 formed by oocyte genes showed mostly negative correlations with outerCC genes in clusters 2 ($\overline{bicor}$= − 0.19), 3 ($\overline{bicor}$= − 0.4), 7 ($\overline{bicor}$= − 0.32) and 1 ($\overline{bicor}$4 = − 0.12) but presented a balanced distribution of positive and negative correlations with cluster 9 ($\overline{bicor}$= 0.04) (Additional file 1: Figure S8a). The correlations formed by genes expressed in oocytes and innerCCs mostly showed negative associations between oocyte genes in cluster 2 and innerCC genes in clusters 2, 4 and 11 ($\overline{bicor}$= − 0.6, $\overline{bicor}$= − 0.46, and $\overline{bicor}$= − 0.46, respectively). By contrast, clusters 1 and 4 formed by oocyte genes mostly showed positive correlations with clusters 2, 4 and 11 formed by innerCC genes ($\overline{bicor}$= 0.58, $\overline{bicor}$= 0.53, $\overline{bicor}$= 0.5, $\overline{bicor}$= 0.39, $\overline{bicor}$= 0.29, $\overline{bicor}$= 0.3, respectively, Additional file 1: Figure S8b). These results highlight patterns of co-expression that potentially resemble the functional regulatory roles between oocytes and cumulus cells.

The network formed from the co-expression results highlighted genes that are likely regulatory hubs between oocytes and cumulus cells. Among the interactions between oocytes and outerCCs, *FHOD3*, *SUGP2*, *ZFP36*, *SRRD*, *ANKRD10*, *RRP8*, *NOL7* and *HOOK2* expressed in oocytes, *PAXIP1* expressed in outerCCs, and *CEBPG* expressed in both oocytes and outerCCs were the top ten genes with the highest degrees of connectivity (Fig. 4c). Among the interactions between oocytes and innerCCs, the *GFER*, *MRPL57*, *GPR137B*, *MAP1LC3A*, *MIER1*, *MAGIX*, *COMMD9*, *C4orf48*, *A1BG* and *HOXD4* genes expressed in oocytes presented the highest degrees of connectivity (Fig. 4d). These results suggest the existence of key genes with major regulatory functions in COCs.

## Discussion

The understanding of how cells interact within a micro-environment is key for the comprehension of a physiological process at the systems levels. The complexity of the molecular interactions between the oocyte and the surrounding cumulus cells in the follicular microenvironment during folliculogenesis has yet to be fully appreciated. In this study, we diligently sampled the oocytes and surrounding cumulus cells of single COCs and generated RNA sequencing data from three sample types from multiple COCs. We used this dataset to gain the following insights into important factors in the communication between oocytes and cumulus cells: (i) cumulus cells positioned closer to or farther from the oocyte are likely to have a distinct interaction with the oocyte; (ii) similarly to the reported for somatic cells in humans [27], there are several potential ligands and receptors that can mediate regulatory signaling between oocytes and cumulus cells; and (iii) the association between the transcript levels of genes expressed in oocytes and cumulus cells strongly indicates that the interaction between oocytes and cumulus cells is partly modulated by gene regulatory mechanisms. The underlying mechanisms of gene regulation and how the cross-modulation of transcript levels contributes to the fate of antral follicles currently remain unknown.

The oocyte accumulates a rich variety of transcripts and proteins through folliculogenesis. The results of co-expression analyses in oocytes showed that coordinated regulatory mechanisms drive the transcription and accumulation of 2222 gene products. Our results highlight dozens of genes with transcriptional regulatory functions (*N* = 69) or functions related to translation (*N* = 54). These genes likely play central roles in the modulation of embryo genome activation [28].

The mechanisms that control gene expression in cumulus cells during the growth of antral follicles are not yet understood. Nevertheless, our results showing coordinated expression of several genes related to “translation” and “oxidation-reduction process” in cumulus cells are indicative that such genes are regulated by gonadotropins [29, 30] or insulin [29]. These results provide support for hormonal regulation as a mechanism that modulates gene expression in cumulus cells. Of note, the distinct patters of gene co-regulation between innerCCs and outerCCs corroborates with previous findings of a greater number of apoptotic cells in the outer layers of cumulus compared to the cells closer to the oocyte [31].

The signaling between cumulus cells and oocytes is central to folliculogenesis. We must ponder, however, that limitations on PPI databases and even on our detection of gene transcripts may have limited the identification of critical interactors in COCs. Furthermore, experimental validation should provide empirical evidence of the interactions presented on this study. Nonetheless, the integration of transcriptome and proteomics datasets provided a genome-wide view of ligand-receptor or receptor-receptor potentially interacting in the COCs.

Our results showed that hundreds of possible ligand-receptor pairs can transduce paracrine signaling. Notably, several genes that are only expressed in oocytes encode ligands that are functionally related to “axon guidance,” while on the other side of the zona pelucida, cumulus cells express several genes involved in cell adhesion. It is possible that the oocyte uses strategies similar to those described for the nervous system [32] to guide the formation of transzonal cellular projections [10, 33] to establish close contact between the oocyte and cumulus cells from the corona radiata. At the same time, the cumulus cells are likely responsible for the signaling required to establish and sustain cellular adhesion with the oocyte membrane [34].

The functional analysis of ligands and receptors indicated that signaling between the oocyte and cumulus cells also regulates gene transcription. Our experimental design allowed us to further explore the possibility that oocytes regulate gene expression in cumulus cells and vice versa. Our results showed that the transcript levels of several hundred genes expressed in oocytes present linear co-variation with genes expressed in cumulus cells. Furthermore, the results indicate functional organization of a select group of co-expressed genes. Such cross-cellular transcriptional regulation could be modulated by paracrine signaling [35], as we identified several potential ligand-receptor pairs that may be formed as well as transfer of small molecules through gap junctions [36] and transport of mRNAs between cumulus cells and oocytes [10]. These findings provide strong evidence that a complex gene regulatory network between oocytes and cumulus cells regulates the maturation of COCs in antral follicles.

## Conclusion

We determined that several hundreds of genes present co-expression within compartments in a cumulus -oocyte complex. The major differences between oocytes and the surrounding cumulus cells include but are not limited to regulation of transcription, and those genes are likely responsible for distinct physiological specialization between cumulus and oocytes. Despite their different functional roles, oocytes and cumulus cells are dependent on each other’s signaling to progress through folliculogenesis. The paracrine signal between the oocyte and cumulus cells can be conducted by hundreds of putative ligand-receptor pairs is a potential venue for transcriptional regulation between oocyte and cumulus cells. The significant correlated expression of thousands of genes in oocytes and cumulus cells is a strong genome-wide evidence that supports the occurrence of a gene regulatory networks between oocyte and cumulus cells. Our findings show that the interaction between oocyte and cumulus cells is much more complex than the exchange of metabolites and involves gene regulation.

## Methods

### Sample collection from single COCs

Ovaries were obtained from *Bos taurus* cows from a commercial slaughter house (Brown Packing, SC). No live animals were handled specifically for this study and ovaries were handled postmortem, thus the study was not submitted for approval by the institutional ethics committee at Auburn University.

Upon removal from the animals, ovaries were placed in a 0.8% saline solution and transported to the laboratory. Ovarian follicles measuring between 3 and 8 mm in diameter were aspirated with an 18-gauge needle, and the aspirates were transferred to sterile 50 ml conical tubes. COCs were then selected from the follicular fluid and washed in TCM-199 medium supplemented with 0.42 M sodium bicarbonate (Macron), 0.02 M Hepes (Sigma-Aldrich), 10% (v:v) fetal bovine serum (Seradigm), 0.05 g/ml gentamicin (Amresco), 0.022 g/ml pyruvate (Acros Organics) and 1× Glutamax (Gibco). COCs were selected based on morphological characteristics indicative of a greater developmental potential [21, 37]. The obtained COCs containing oocytes with homogeneous cytoplasm surrounded by more than five layers of cumulus cells were used in our research. This strategy was adopted to eliminate COCs collected from degenerating follicles, so that the RNA of healthy COCs would be profiled.

The selected COCs were individually transferred to a 5 μl droplet of 1× PBS 0.02% BSA (Akron). The COCs were then transferred to a droplet containing 1× Trypsin (HyClone Laboratories), and the outer layer of CCs was removed through gentle pipetting. These outer cumulus cells were collected, flash frozen and stored at − 80 °C. The remaining COC (the oocyte and approximately two/three layers of cumulus cells) was then washed in fresh 1× PBS 0.02% BSA and then transferred to a droplet of 1× Trypsin. The remaining layers of cumulus cells were removed by gentle pipetting. The denuded oocytes were transferred twice to fresh droplets of 1× PBS 0.02% BSA to avoid carryover of cumulus cells. A schematic of the sample handling with representative images of the samples is depicted in Additional file 1: Figure S9. Five oocytes were examined via fluorescence microscopy to verify that no CCs remained attached to the oocyte (not snap frozen for RNA analysis, Additional file 1: Figure S10). The inner cumulus cells were collected, snap frozen in liquid nitrogen, and stored at − 80 °C. The oocyte was then collected with using minimal 1× PBS 0.02% BSA solution and snap frozen in liquid nitrogen. All samples were stored at − 80 °C until use for cell lysis.

### Library preparation and RNA sequencing

For each of the 16 COCs, we prepared libraries for the oocyte, innerCC and outerCC cells. We extracted RNA from cumulus cells using the guanidine thiocyanate-phenol chloroform procedure [38, 39] (TRIzol reagent, Thermo Fisher), with the addition of 0.5 μl of GlycoBlue Coprecipitant (Thermo Fisher) as the RNA carrier [40]. We eluted the RNA from CCs in 4 μl of a solution containing dNTPs and oligo-dT_30_VN and proceeded with polyA+ whole transcriptome amplification using the SMART-seq2 protocol [41, 42] for cDNA amplification. For the oocytes, we added 2 μl of lysis buffer (20 IU/μl RNase inhibitor (Amresco), 0.2% Triton X-100 (Amresco)) to the tube and proceeded with polyA+ whole transcriptome amplification using the SMART-seq2 protocol [41, 42] for cDNA amplification. The samples were subjected to 16 cycles of PCR amplification. For all samples, we used 1 ng of amplified cDNA for library preparation with the Nextera XT DNA library preg Kit (Illumina, Inc.), as per the procedures described in the SMART-seq2 protocol [41, 42]. The libraries were quantified using a Qubit 3.0 fluorometer (Thermo Fisher) and assayed for quality assessment on a 2100 Bioanalyzer System (Agilent). The libraries were sequenced at the Genomic Services Lab at HudsonAlpha in Huntsville, AL, on HiSeq2500 equipment (Illumina, Inc.) to produce paired-end reads of 100 nucleotides in length.

### Estimation of gene expression levels and principal component analysis

The libraries were aligned against the *Bos taurus* genome, UMD3.1, downloaded from Ensembl [43] using the STAR (v2.5.2) [44] aligner. Only reads showing a unique match to the genome and less than five mismatches were further filtered to eliminate duplicates with picard (v2.5, http://broadinstitute.github.io/picard/). The bam files containing non-duplicated reads were employed as input for Cufflinks (v.2.2.1), together with Ensembl gene annotation [45] UMD3.1.87, to obtain FPKM data [46]. Genes were subjected to analytical procedures if FPKM > 0.5 in eight or more cells of each cell type (oocytes, innerCCs and outerCCs). All statistical analyses were conducted in R software [47]. Codes are available from the corresponding author upon request.

To identify the main sources of variation in the dataset, we employed the FPKM values as the input for principal component analysis using the FactorMiner R package [48]. The significance of the principal components was obtained with the Seurat package [49] via a permutation test, after 1000 randomized samplings [50].

### Correlation network analysis of gene expression within sample types

We performed weighted gene correlation network analysis each of the sample types (oocytes, innerCCs and outerCCs) using the WCGNA package [51]. For each of the three sample types, we used log_2_(FKPM+ 1) values to calculate signed adjacency, followed by the calculation of topological overlap similarity to identify patterns of interconnectivity among genes [51]. The topological overlap matrix (TOM) was converted into a distance matrix (1-TOM) for clustering, using the average method and the Euclidian distance, with the flashClust package [52]. GO [53] enrichment was performed for co-expression modules by cutting the tree at different heights, and the most representative cutting is presented in the main or Supplementary figures.

### Testing gene sets for GO enrichment

The GO [53] enrichment of the gene lists was tested using the genes expressed in the corresponding sample types (oocytes, innerCCs or outerCCs) as the background. We obtained GO annotations and transcript lengths from BioMart [54] and used the GOseq package [55] to estimate enrichment significance via the Wallenius approximation method [55]. We used FDR for multiple testing under dependency [56] to adjust *P*-values. GO terms with an FDR < 0.2 were inferred to be statistically significant.

### Comparison of gene expression between oocytes and cumulus cells

The non-duplicated reads were subjected to counting according to Ensembl gene annotation [45] UMD3.1.87 using HTSeq (v. 0.6.1) software [57]. We employed the raw read count to compare gene expression levels between samples with the packages edgeR [58], using the TMM normalization [59] and DeSeq2 [60] using sample- and gene-specific normalization factors as described elsewhere [60]. The model considered the two cell types being compared (oocyte vs. innerCC, oocyte vs. outerCC) and the collection batch (batch = 1, 2). We inferred that differential gene expression existed if the Benjamini-Hochberg [61] FDR was < 0.01 in the results obtained using both packages.

### Validation of differential gene expression by RT-qPCR

We selected five genes that were upregulated in oocytes compared with cumulus cells for validation of the RNAseq results (Additional file 3: Table S7). The genes were selected according to their previously recognized roles in oocyte acquisition of developmental competence.

We collected three pools of oocytes and cumulus samples, using 35–40 COCs for each pool. Total RNA was extracted using the TRIzol reagent, and RNA equivalent to the content of five oocytes or cumulus cells from five COCs was used for cDNA amplification using the SMART-Seq2 procedures. Amplified cDNA was purified and used as template for qPCR.

qPCR was performed using 0.5 ng of amplified DNA, 0.1 nM of specific primers (Additional file 3: Table S13) and Perfecta SYBR Green FastMix (Quanta Biosciences), in a final reaction of 10 μl. The reactions were assayed in Roche Light Cycler 480 equipment (Roche), with pre-incubation at 95 °C for 1 min, followed by 40 cycles of 95 °C for 15 s and 60 °C for 45 s. A melting curve was subsequently generated using the thermocycler’s default parameters to validate primer specificity.

We used the *glyceraldehyde 3-phosphate dehydrogenase* (*GAPDH*) gene as the internal control to normalize the expression levels of target genes (primers: TGGTGAAGGTCGGAGTGAAC, ATGGCGACGATGTCCACTTT). Primers were designed using Primer-BLAST [62] and are described on Additional file 3: Table S7. The PCR efficiency was estimated for each primer set with LinRegPCR [63], and relative expression values were calculated using the method described for primers with different amplification efficiencies [64]. The results shown in Additional file 3: Table S7 are expressed as the fold changes in oocytes relative to those in cumulus cells. We assessed the significance of the fold changes by comparing the averages ΔC_T_ [65] values with Student’s t-test [66].

### Identification of putative ligands and receptors in cumulus-oocyte complexes

First, we produced a comprehensive PPI database. We downloaded the databases from BioGRID [67], MINT [68], DIP [69] and IntAct [70] and filtered them to retain the interactions from cow, human, mouse, pig, rabbit and rat. The gene or protein identifiers were then converted to Ensemble gene identifiers using Entrez [71] gene-to-Ensembl mapping for BioGRID or UniProt-SwissProt [72]-to-Ensembl mapping for MINT, DIP and IntAct. The mapping of gene homology between each of the selected species and cow was obtained from the BioMart database [54, 73] (accessed on 03/09/2017), and the database was further filtered to eliminate duplicate interactions. We ultimately retained 247,064 putative PPIs.

Next, we used BioMart [73] (accessed on 03/23/2017) to annotate the genes with GO terms. We retained the pairs that contained one or two gene identifiers annotated with either the term “receptor complex” or “receptor activity”, which are the broadest terms associated with receptors in the GO tree structure. Here we designated “ligand” a protein that binds to a protein that is a receptor or is part or a receptor complex. We then mapped the gene identifier to the three transcriptome datasets (oocyte, innerCC and outerCC) to identify potential protein-protein pairs enriched for ligands and receptors in the cumulus-oocyte complexes. We conducted tests of GO enrichment with ligands following the procedures described above, and we drew circle plots with the circlize package [74].

### Functional annotation of highly variable genes

To achieve robust functional annotation of variable genes, we retained only those genes that were detected in all 16 oocytes (*N* = 6701) or all 16 outerCC samples (*N* = 7168). We did not conduct this analysis for innerCCs because there were only a few hundred genes expressed in all 16 samples. For each gene, we calculated the coefficient of variance according to$CV=\sqrt{\left({\mathit{\exp}}^{\left({x}^2\right)}\right)-1}$, where *x* is the sample standard deviation of log_2_(FPKM+ 1) for each gene. Then, we carried out GO enrichment tests for the genes with highly variable expression.

### Calculation of the pairwise correlation of transcript levels between genes expressed in oocytes and surrounding cumulus cells

For each of the 16 COCs collected, we sampled the oocyte, innerCCs and outerCCs. We leveraged this sampling structure to calculate the biweighted correlation (bicor [25, 52]) between the expression levels of genes expressed in oocytes and the surrounding cumulus cells according to the following framework. Let *x*_*kj*_ be the log_2_(FPKM+ 1) value of gene *k*_(1, …, *n*)_ in the oocyte, *y*_*lj*_ the log_2_(FPKM+ 1) value of gene *l*_(1, …, *n*)_ in the innerCCs, and *z*_*mj*_ the log_2_(FPKM+ 1) value of gene *m*_(1, …, *n*)_, in the COC *j*_(1, .., 16)_. We calculated ${bicor}_{\left({x}_{kj},{y}_{lj}\right)}$ and ${bicor}_{\left({x}_{kj},{z}_{mj}\right)}$ using the “bicor()” function [52] in R as the coefficient of the association of expression levels between genes transcribed in different cells.

The statistical significance of the coefficients was estimated using eFDR [75, 76]. We disrupted the connection of a COC linking each pair of samples (i.e., oocyte and innerCCs) by permuting the COC index *j*_(1, .., 16)_ from the oocyte samples and calculated ${bicor}_{\left({x}_{kj},{y}_{lj}\right)}^{0b}$ and ${bicor}_{\left({x}_{kj},{z}_{mj}\right)}^{0b}$ for each gene pair for 10,000 permutations (B) with no replacements (for the oocyte and innerCCs or the oocyte and outerCCs independently). Then, eFDR for ${bicor}_{\left({x}_{kj},{y}_{lj}\right)}$ was estimated as $\sum \limits_{b=1}^B\frac{\#\left(k:\left|{bicor}_{\left({x}_{kj},{y}_{lj}\right)}^{0b}\right|\ge \left|{bicor}_{\left({x}_{kj},{y}_{lj}\right)}\right|,\kern0.75em k=1,\dots, \left(k\times l\right)\right)+1}{\left(k\times l\times B\right)+1}$, as described in the literature [76]. Because this method can be computationally intensive, we calculated eFDR for specific values of biweighted correlation (Additional file 1: Figure S7a).

### Functional analysis of the co-expression network between oocytes and the surrounding cumulus cells

We conducted the network analyses with genes that showed highly significant (eFDR< 1.8 × 10^− 5^) and strong correlations (|bicor| > 0.85, N_oocyte_ = 819 and N_innerCC_ = 4569; N_oocyte_ = 1781 and N_outerCC_ = 3187). Here, we describe the application of the method for oocytes and outerCCs, but this method was also applied for oocytes and innerCCs. First, we calculated a distance matrix ($1-{bicor}_{\left({x}_{kj},{y}_{lj}\right)}$) and used it as the input for clustering (average method and Euclidian distance) of the two dimensions (i.e., genes from oocytes in rows and genes from outerCCs in columns). We cut each tree at different heights and tested the clusters for enrichment of biological functions from the GO database (see description above). We used the ComplexHeatmaps package [77] to draw annotated heatmaps and Cytoscape [78] software to visualize the networks. The same approach was used for oocytes and innerCCs.

## Additional files

Additional file 1: **Figures S1-S10.** Supplementary Figures to Functional signaling and gene regulatory networks between the oocyte and the surrounding cumulus cell. (PDF 12330 kb)

Additional file 2: List of genes specifically expressed in oocytes and cumulus cells. Four spreadsheets containing genes specifically expressed in oocytes, inner CCs, outerCC and CCs, average FPKM, and Ensembl annotation. (XLSX 314 kb)

Additional file 3: **Tables S1-S13.** Supplementary Tables to Functional signaling and gene regulatory networks between the oocyte and the surrounding cumulus cell. (XLSX 110 kb)

Additional file 4: Annotation of the 14,011 Ensembl pairs of genes corresponding to potential ligand-receptor or receptor-receptor interactions between oocytes and cumulus cells. Table containing Ensembl identifiers, expression levels and annotation of genes corresponding to potential ligand-receptor or receptor-receptor interactions between oocytes and cumulus cells. (XLSX 1381 kb)

## Abbreviations

bicor: biweight correlation
CC: Cumulus cells
COC: Cumulus oocyte complex
CV: Coefficient of variation
eFDR: empirical false discovery rate
FPKM: Fragment per kilobase per million
GO: Gene ontology
N: number
PPI: Protein-protein interaction
qPCR: quantitative polymerase chain reaction
TOM: Topological overlapping matrix
