# Supplementary material for: Functional signaling and gene regulatory networks between the oocyte and the surrounding cumulus cells
Source: BMC Genomics. 2018 May 10;19:351. doi: 10.1186/s12864-018-4738-2 (PMC5946446; doi:10.1186/s12864-018-4738-2)
Supplement: Supplementary file 1 — Figures S1-S10. Supplementary Figures to Functional signaling and gene regulatory networks between the oocyte and the surrounding cumulus cell. (PDF 12330 kb) [file 12864_2018_4738_MOESM1_ESM.pdf]

Supplementary Figures and Tables to Functional signaling and gene regulatory networks between the oocyte and the surrounding cumulus cells.

Fernando H. Biase*, Katelyn M. Kimble

Department of Animal Sciences, Auburn University, Auburn, AL

*Corresponding author

Address: 559 Devall Dr, Auburn, AL 36849

Email: fbiase@auburn.edu

Phone: 334 844 1680

Fax: 334-844-1519

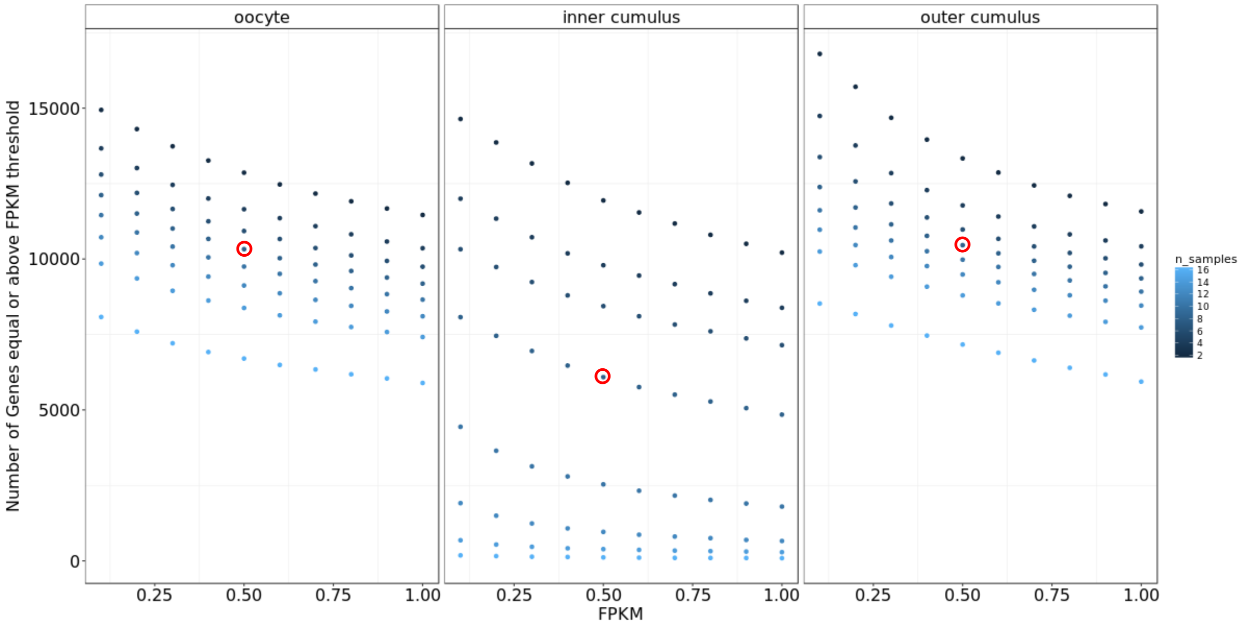

Fig S1. Number of genes detected by RNA-seq according to different thresholds. Red circles mark the number of genes used for analytical procedures.

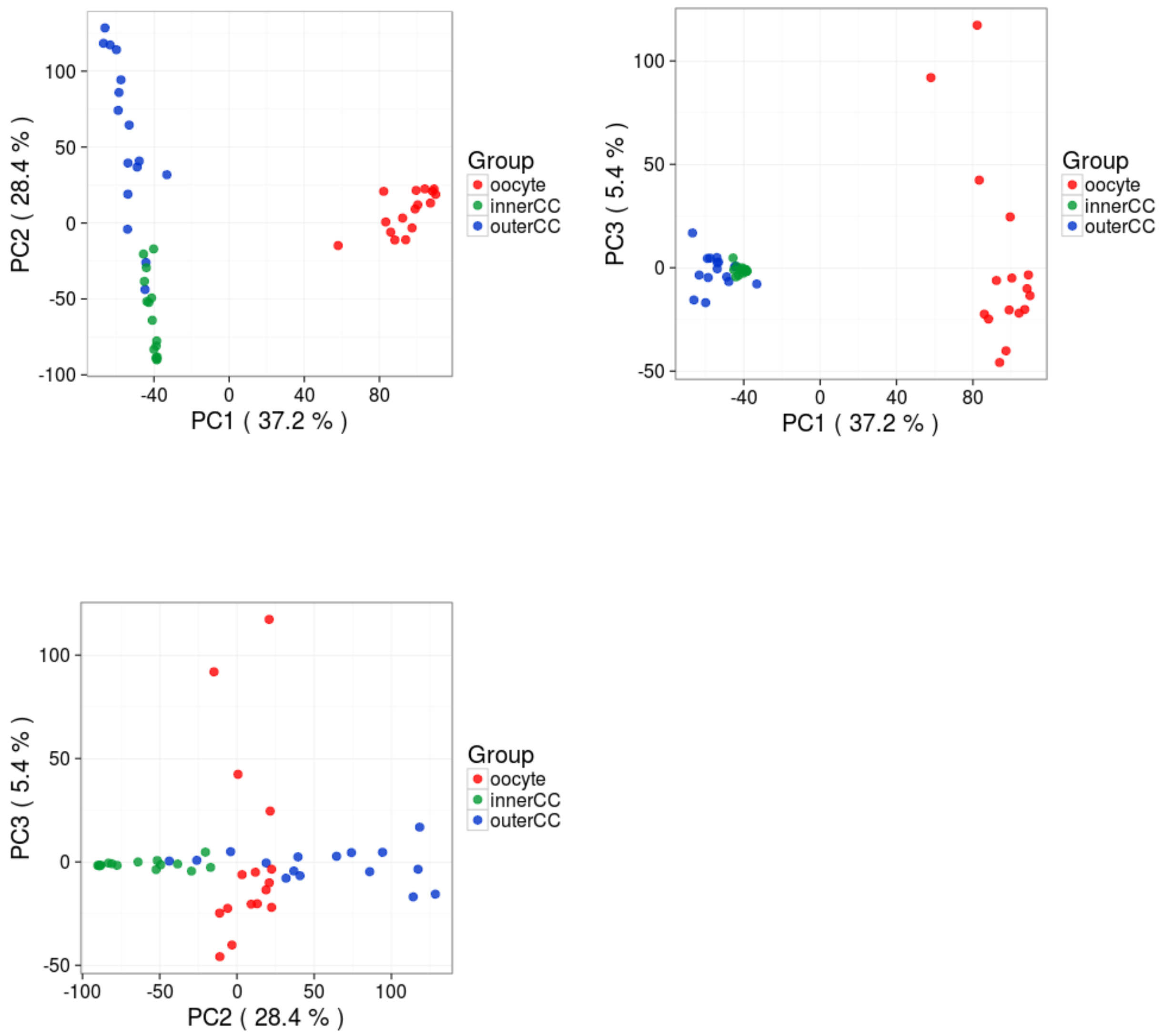

Fig S2. Principal component analysis of the samples collected.

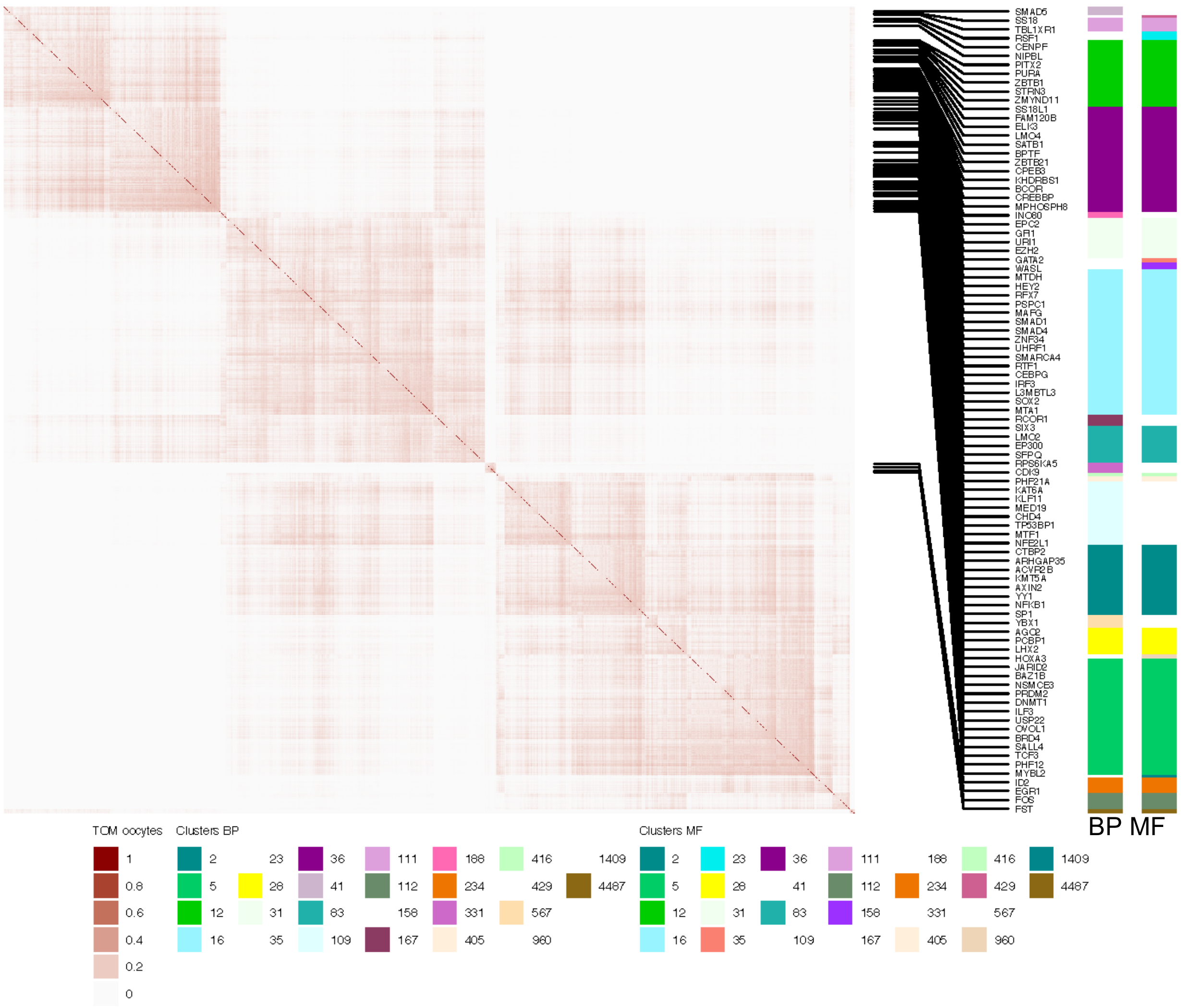

Fig. S3. Topological overlap of transcript levels for 2,222 genes expressed in oocytes. Genes related to regulation of transcription are annotated. The two vertical bars on the right-hand side of the panel annotate clusters with enrichment for gene ontology biological processes (BP) and molecular functions (MF).

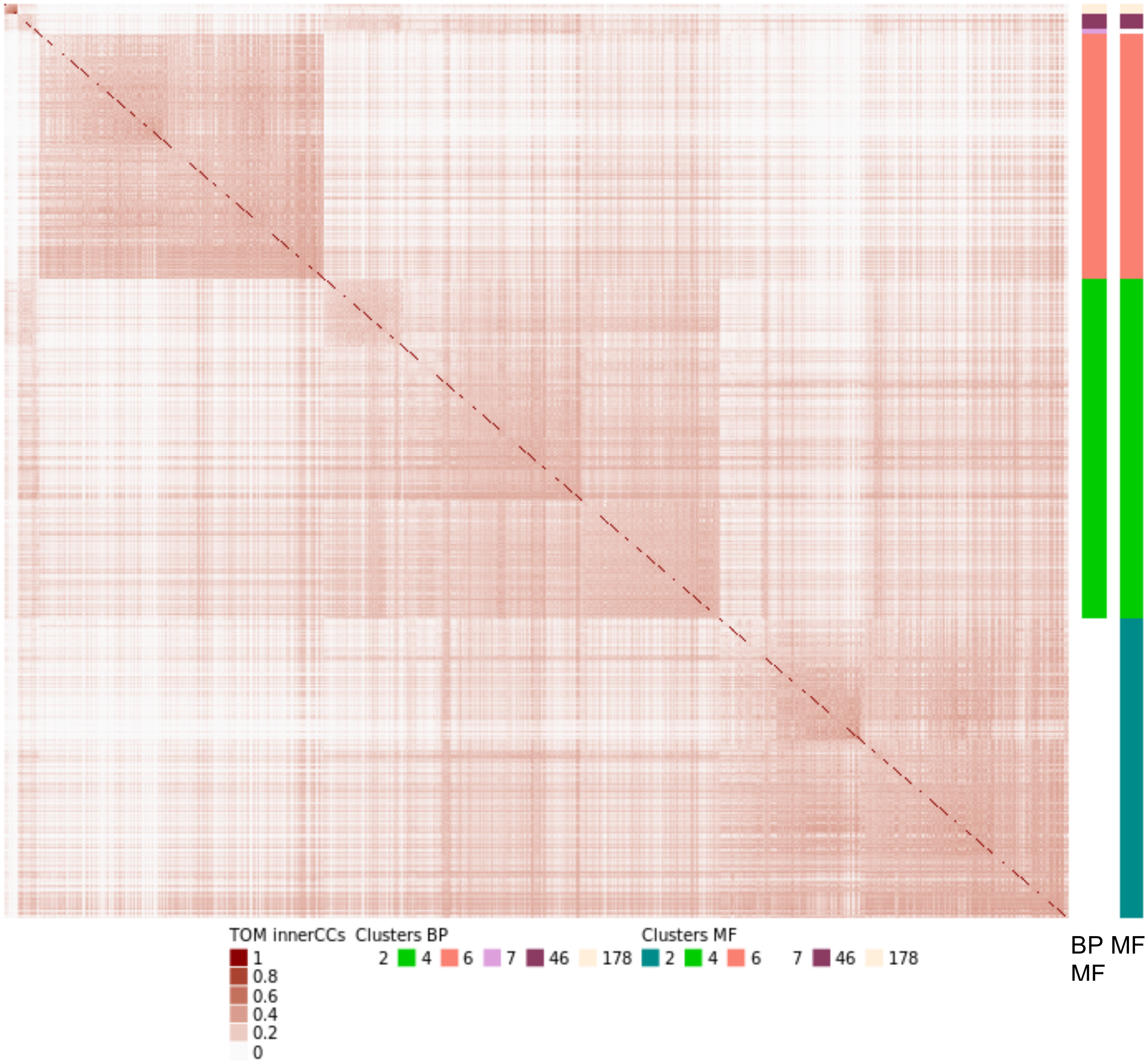

Fig. S4. Topological overlap of transcript levels for 1,222 genes expressed in cumulus cells forming the corona radiata of the cumulus-oocyte complex. The two vertical bars on the right-hand side of the panel annotate clusters with enrichment for gene ontology biological processes (BP) and molecular functions (MF).

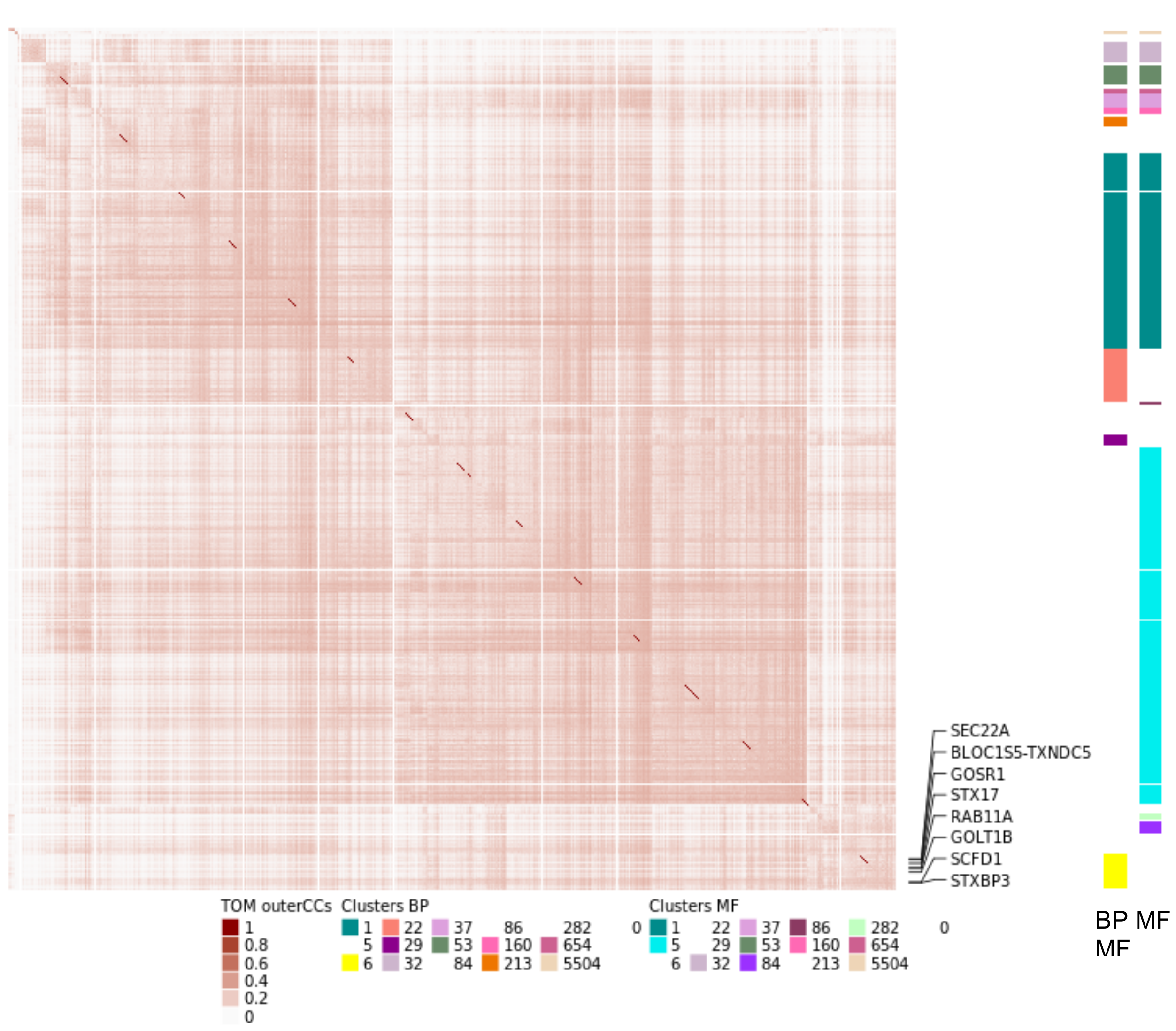

Fig. S5. Topological overlap of transcript levels for 3,990 genes expressed in cumulus cells on the periphery of the cumulus oocyte complex. Genes composing vesicle-mediated transport are annotated on the heatmap. The two vertical bars on the right-hand side of the panel annotate clusters with enrichment for gene ontology biological processes (BP) and molecular functions (MF).

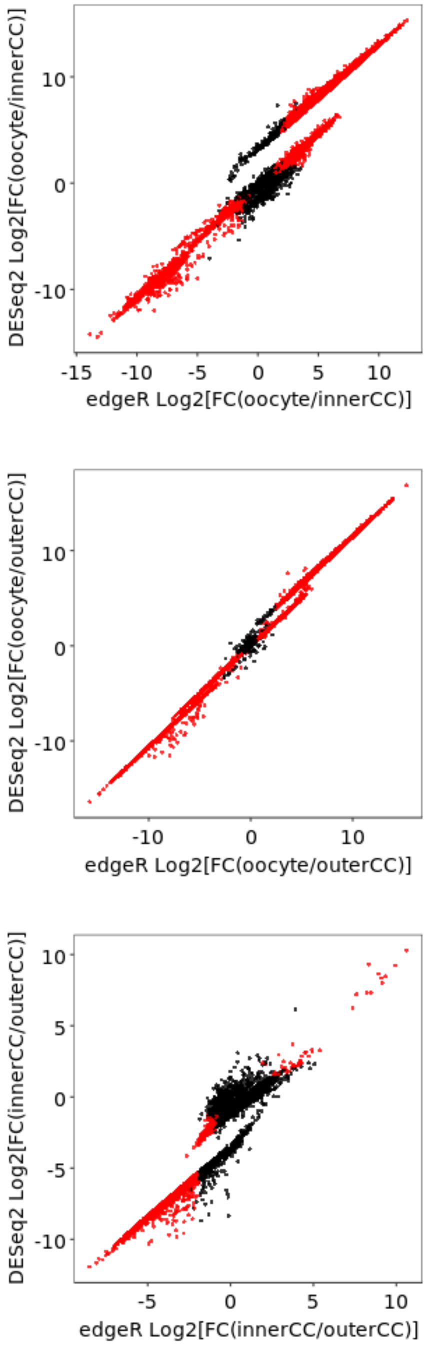

Fig. S6. Comparative results obtained from two algorithms used for pairwise contrast of gene expression levels among oocyte, innerCC and outerCC samples. Red dots represent differentially expressed genes between two groups (FDR<0.01).

a

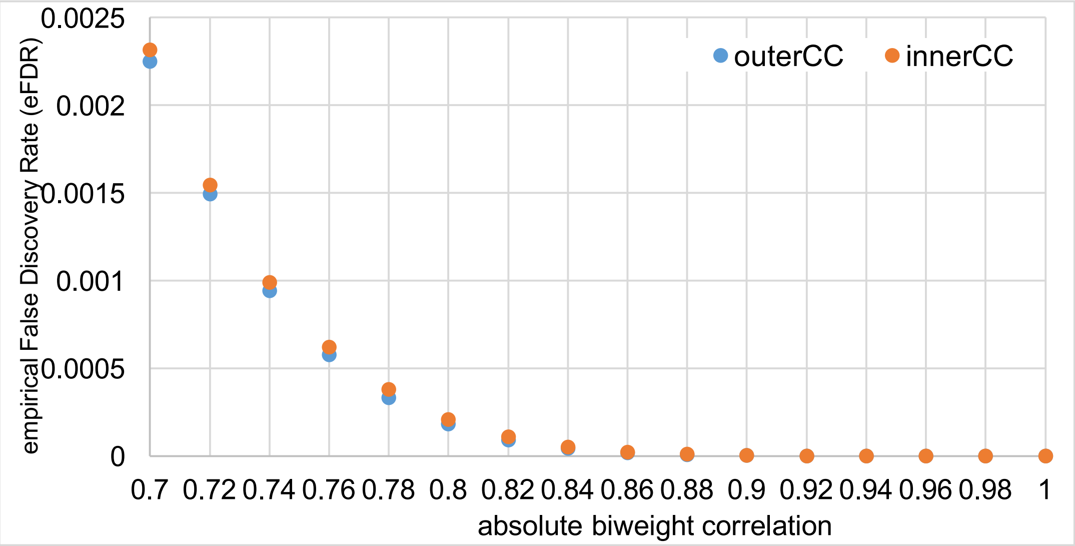

c

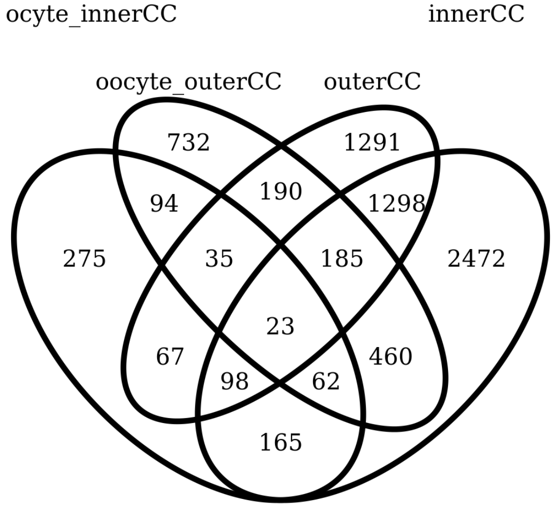

b

bicor	eFDR threshold	N Pairs	N genes		eFDR threshold	N Pairs	N genes		Fisher's exact test[a]
			oocyte	outerCC			oocyte	innerCC	
-0.75	<0.00058	69,206	4,857	8,619	< 0.0006	86,296	1,119	5,925	<2.2e-16
-0.80	0.00018	17,621	2,530	5,128	0.00021	29,348	525	5,248	<2.2e-16
-0.85	<1.86E-05	2,828	856	1,569	<2.26E-05	6,372	196	3,027	<2.2e-16
-0.90	1.98E-06	193	125	151	3.09E-06	545	37	505	<2.2e-16
-0.95	<1.39E-08	2	2	2	<3.50E-08	0	0	0	-
0.75	<0.00058	88,810	4,694	9,376	< 0.0006	418,170	2,529	5,951	3.29e-08
0.80	0.00018	23,753	2,514	6,274	0.00021	142,794	1,419	5,530	<2.2e-16
0.85	<1.86E-05	4,206	940	2,237	<2.26E-05	36,471	623	4,512	<2.2e-16
0.90	1.98E-06	326	180	264	3.09E-06	6,520	163	2,620	<2.2e-16
0.95	<1.39E-08	4	4	4	<3.50E-08	453	11	416	5.979e-05

a. Fisher's exact test for the number of oocyte and cumulus cells genes observed at each threshold level.

Fig. S7. Descriptive properties of the co-expression between oocytes and cumulus cells. (a) Estimates of eFDR from the correlations obtained from scrambled oocyte data and outerCCs and innerCCs data. (b) Distribution of genes and pairs of genes according to different thresholds. (c) Overlapping of genes forming significant correlation links at |bicor|>0.85 and eFDR <1.86E-05.

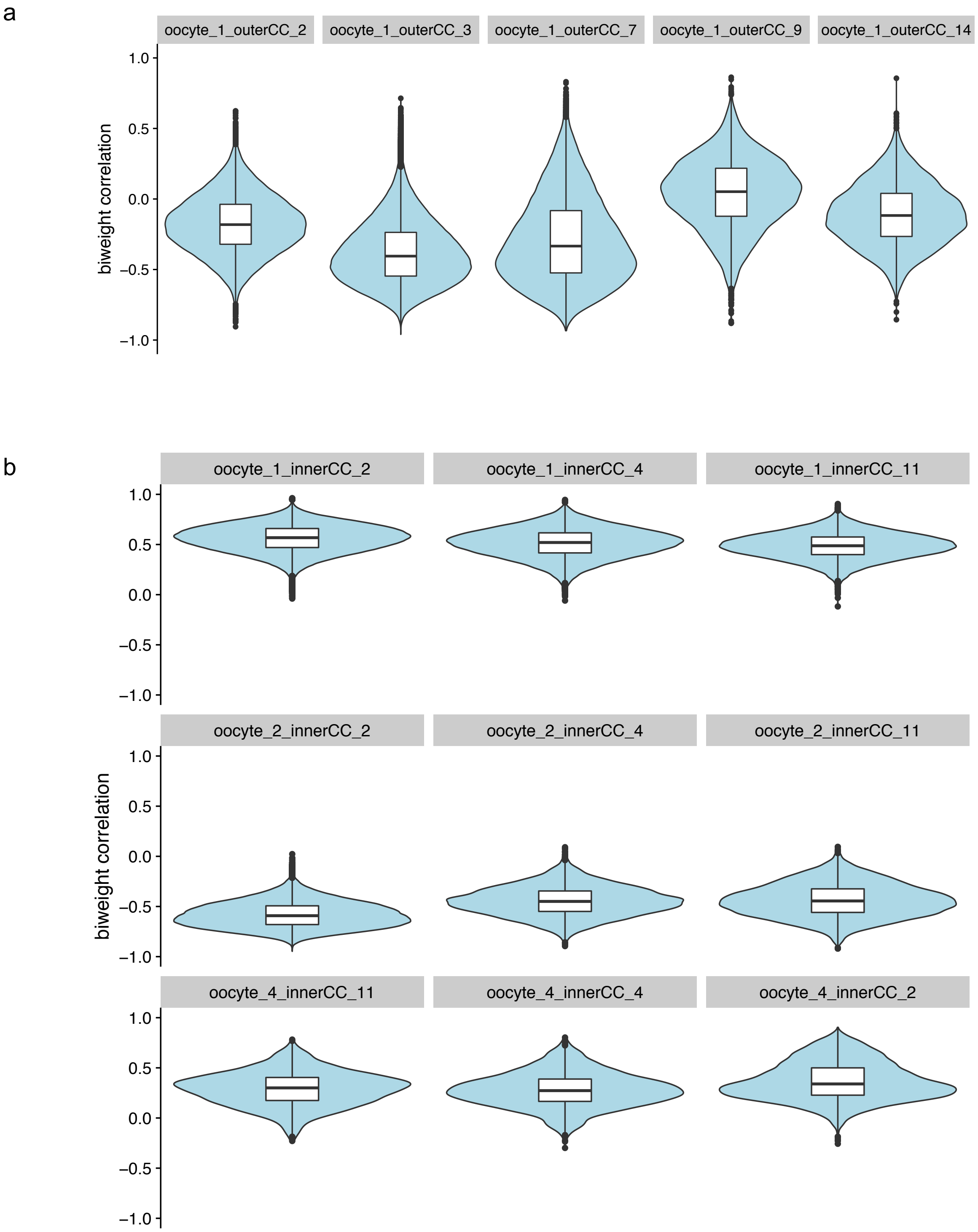

Fig. S8. Average pair-wise gene correlation between transcript levels in oocytes and outerCCs (a) or innerCCs (b). Each violin plot represents a block of co-expressing genes delimited by their clusters (see Fig. 4a, a), and are named according to the cluster in oocyte and cluter in cumulus cells (i.e.: oocyte_4_innerCC_2, means cluster 4 formed by oocytes genes and cluster 2 formed by innerCC genes).

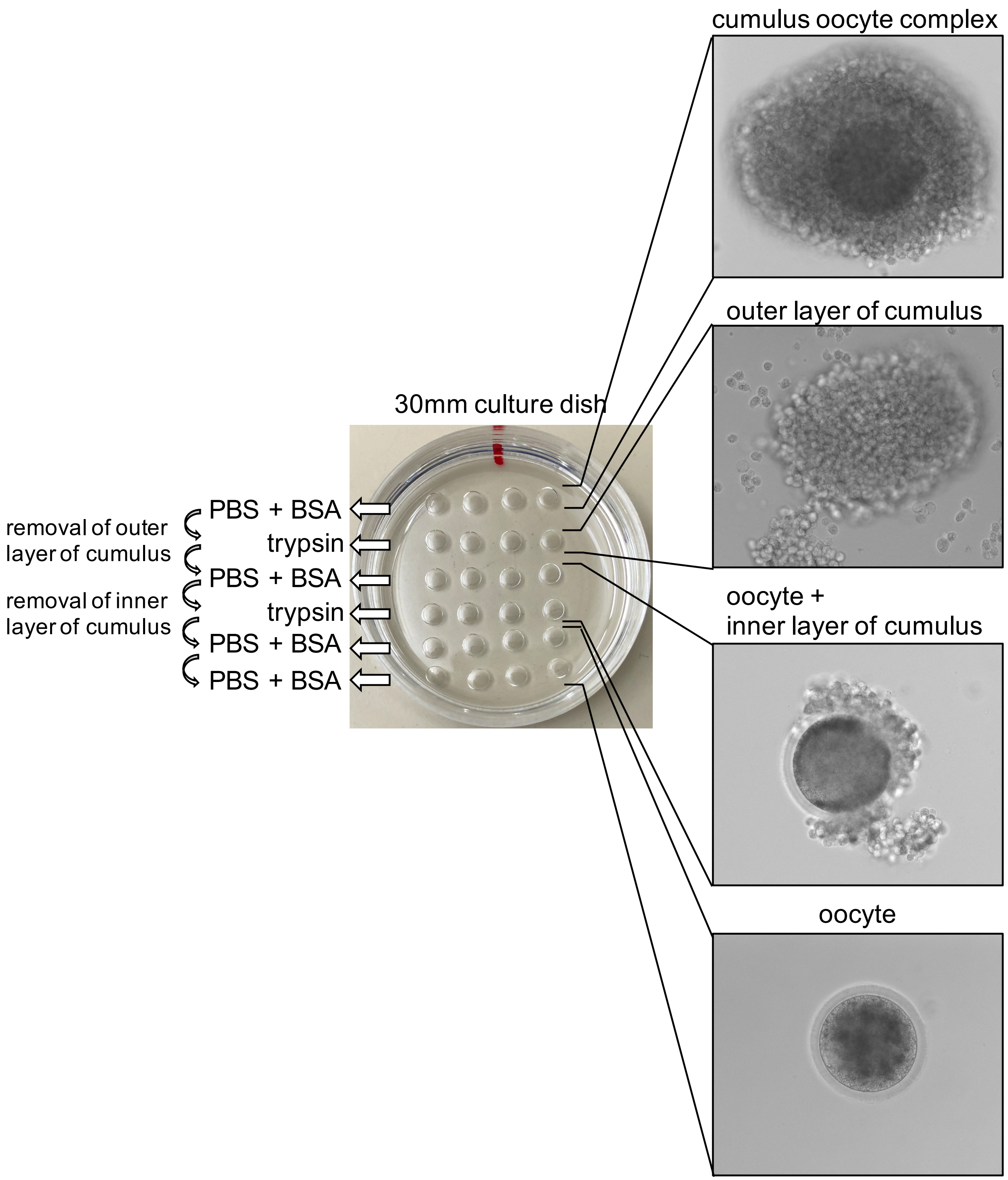

Fig. S9. Schematics of the sample collection. Please, see Methods section for detailed description of the sample collection.

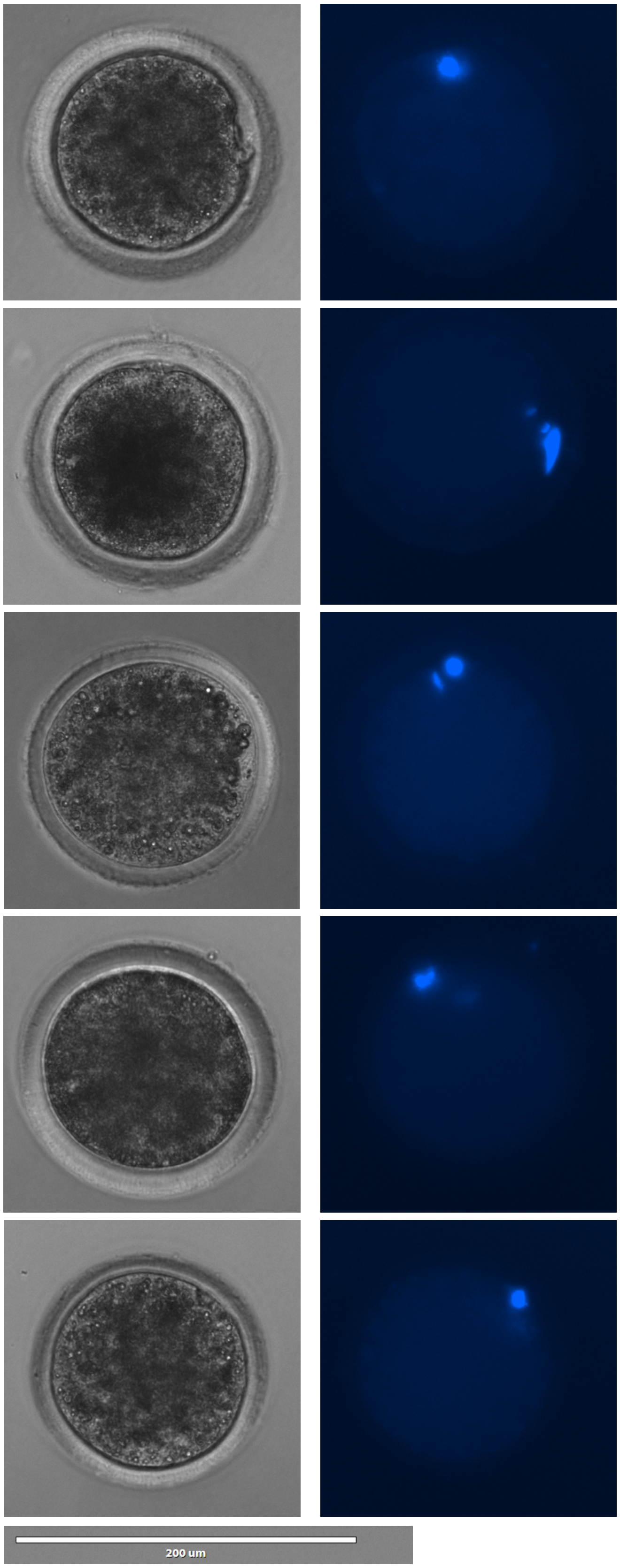

Fig. S10. Representative oocytes free of cumulus cells collected for RNA-sequencing. Left panel: Bright field, Right panel DAPI, the scale bar (200μm) is used for all pictures.
